# Multiparametric MRI–based radiomic models for early prediction of response to neoadjuvant systemic therapy in triple-negative breast cancer

**DOI:** 10.1038/s41598-024-66220-9

**Published:** 2024-07-12

**Authors:** Rania M. Mohamed, Bikash Panthi, Beatriz E. Adrada, Medine Boge, Rosalind P. Candelaria, Huiqin Chen, Mary S. Guirguis, Kelly K. Hunt, Lei Huo, Ken-Pin Hwang, Anil Korkut, Jennifer K. Litton, Tanya W. Moseley, Sanaz Pashapoor, Miral M. Patel, Brandy Reed, Marion E. Scoggins, Jong Bum Son, Alastair Thompson, Debu Tripathy, Vicente Valero, Peng Wei, Jason White, Gary J. Whitman, Zhan Xu, Wei Yang, Clinton Yam, Jingfei Ma, Gaiane M. Rauch

**Affiliations:** 1 Department of Breast Imaging, The University of Texas MD Anderson Cancer Center, 1515 Holcombe Blvd, # 1473, Houston, TX 77030 USA; 2 Department of Cancer Systems Imaging, The University of Texas MD Anderson Cancer Center, Houston, TX USA; 3 Department of Imaging Physics, The University of Texas MD Anderson Cancer Center, Houston, TX USA; 4 Koc University Hospital, Istanbul, Turkey; 5 Department of Biostatistics, The University of Texas MD Anderson Cancer Center, Houston, TX USA; 6 Department of Breast Surgical Oncology, The University of Texas MD Anderson Cancer Center, Houston, TX USA; 7 Department of Pathology, The University of Texas MD Anderson Cancer Center, Houston, TX USA; 8 Department of Bioinformatics and Computational Biology, The University of Texas MD Anderson Cancer Center, Houston, TX USA; 9 Department of Breast Medical Oncology, The University of Texas MD Anderson Cancer Center, Houston, TX USA; 10 Department of Surgery, Baylor College of Medicine, Houston, TX USA; 11 Department of Abdominal Imaging, The University of Texas MD Anderson Cancer Center, Houston, TX USA

**Keywords:** Triple-negative breast cancer, Dynamic contrast-enhanced breast MRI, Diffusion-weighted imaging, Neoadjuvant systemic therapy, Treatment response, Radiomic features, Breast cancer, Prognostic markers

## Abstract

Triple-negative breast cancer (TNBC) is often treated with neoadjuvant systemic therapy (NAST). We investigated if radiomic models based on multiparametric Magnetic Resonance Imaging (MRI) obtained early during NAST predict pathologic complete response (pCR). We included 163 patients with stage I-III TNBC with multiparametric MRI at baseline and after 2 (C2) and 4 cycles of NAST. Seventy-eight patients (48%) had pCR, and 85 (52%) had non-pCR. Thirty-six multivariate models combining radiomic features from dynamic contrast-enhanced MRI and diffusion-weighted imaging had an area under the receiver operating characteristics curve (AUC) > 0.7. The top-performing model combined 35 radiomic features of relative difference between C2 and baseline; had an AUC = 0.905 in the training and AUC = 0.802 in the testing set. There was high inter-reader agreement and very similar AUC values of the pCR prediction models for the 2 readers. Our data supports multiparametric MRI-based radiomic models for early prediction of NAST response in TNBC.

## Introduction

Triple-negative breast cancer (TNBC) is defined as negative for estrogen receptor, progesterone receptor, and human epidermal growth factor receptor 2 (HER2)^1,2^. TNBC is an aggressive cancer typically treated with neoadjuvant systemic therapy (NAST)^3^. Among patients with TNBC treated with NAST, those with a pathologic complete response (pCR) have better clinical outcomes^4–6^. Thus, noninvasive prediction of pCR status early in the course of NAST is of high importance. Patients likely to have incomplete or no response (a non-pCR) to NAST can be triaged to clinical trials of novel therapies^7,8^, and patients likely to achieve pCR can continue standard-of-care treatment or qualify for investigational treatment de-escalation^6,9^.

The most accurate diagnostic imaging modality for baseline staging of breast cancer and assessment of breast cancer response to NAST is dynamic contrast-enhanced MRI (DCE MRI)^10–12^. Diffusion-weighted MRI (DWI) with quantitative apparent diffusion coefficient (ADC) complements DCE MRI and can provide additional functional information^13,14^.

Radiomic features from DCE MRI and DWI images, i.e., quantitative features that describe characteristics of tumors and their surroundings and are extracted from MR images by use of computer algorithms^15^, can help predict the response of breast cancer to treatment^15^. DCE MRI radiomic features, such as Haralick gray-level co-occurrence matrix (GLCM)-based features, characterize tumor enhancement heterogeneity, which has been shown to correlate with aggressive growth and poor prognosis of breast cancer and can be helpful for noninvasive prediction of breast cancer response to treatment^16–18^. Several studies have also shown that DWI radiomic features can be used for breast lesion diagnosis^18^, to distinguish between benign and malignant breast lesions^19^, to differentiate TNBC from other breast cancer subtypes^20^, and potentially for predicting breast cancer response to treatment^14,21^.

As DCE MRI and DWI are the most utilized MRI diagnostic sequences, there is interest in investigating the combination of DCE MRI and DWI radiomic features to improve assessment of tumor biology before and during treatment to better guide treatment^22^. Functional MRI techniques included in the multiparametric imaging of breast tumors provide more complete pathophysiologic information about breast cancer^23^ that can improve diagnostic accuracy and assessment of response to NAST^24^.

The purpose of our study was to determine if radiomic signatures based on multiparamteric DCE MRI and DWI images obtained early during NAST can predict pCR in patients with TNBC.

## Results

### Patient characteristics and pCR status

The study included 163 patients with TNBC who underwent NAST and had MRI at baseline (BL) and after 2 cycles (C2) and after 4 cycles (C4) of NAST with an MRI protocol consisting of an axial T2-weighted sequence, a DCE MRI sequence, and a DWI sequence. Seventy-eight patients (48%) had pCR, and 85 (52%) had non-pCR. Data from 109 patients were used as the training set, and data from the other 54 patients were used as the testing set, following a 2:1 training to testing sample size ratio. The training set included 52 (32%) patients with pCR and 57 (35%) patients with non-pCR and the testing set included 26 (16%) patients with pCR and 28 (17%) patients with non-pCR. Patient characteristics are summarized in Table 1

**Table 1:** Characteristics of patients with TNBC who received NAST by pCR status*

Characteristic	Total (N = 163)	Non-pCR (N = 85) (52%)	pCR (N = 78) (48%)	*P* value
Age, median (range), yr	49 (23–78)	50 (31–78)	48 (23–78)	
Histologic type				0.741
Invasive ductal carcinoma	146 (89)	74 (87)	72 (93)	
Metaplastic	11 (7)	7 (8)	4 (5)	
Invasive mammary carcinoma	3 (2)	2 (3)	1 (1)	
Poorly differentiated carcinoma	2 (1)	1 (1)	1 (1)	
Apocrine	1 (1)	1 (1)	0 (0)	
Clinical stage				0.690
I	22 (13)	11 (13)	11 (14)	
II	114 (70)	59 (69)	55 (71)	
III	27 (17)	15 (18)	12 (15)	
T category				0.158
T1	31 (19)	12 (14)	19 (24)	
T2	112 (69)	59 (70)	53 (68)	
T3	18 (11)	13 (15)	5 (7)	
T4	2 (1)	1 (1)	1 (1)	
N category at diagnosis				0.850
N0	108 (66)	55 (65)	53 (68)	
N1	36 (22)	19 (22)	17 (22)	
N2	7 (4)	4 (5)	3 (4)	
N3	12 (8)	7 (8)	5 (6)	
Type of surgery				0.745
Breast-conserving surgery	96 (59)	47 (55)	49 (63)	
Total mastectomy	67 (41)	38 (45)	29 (37)	

*Values in table are number of patients (percentage) unless otherwise indicated.

TNBC, Triple negative breast cancer; pCR, Pathologic complete response; Yr, Year.

### Imaging features associated with pCR status by univariate analysis

Out of 10 first-order radiomic features and 300 GLCM radiomic features, univariate analysis identified 131 imaging features from both DCE MRI and DWI that predicted pCR status with area under the receiver operating characteristics curve (AUC) ≥ 0.7 in both the training and testing sets. Specifically, 25 first-order radiomic (histogram) features from the DCE images (AUC 0.70–0.85 for the training set and 0.70–0.81 for the testing set; Supplementary Table 1) and 106 GLCM features from the DWI images (AUC 0.70–0.79 for the training set and 0.70–0.81 for the testing set; Supplementary Table 2) at both the C2 and C4 time points and changes between C4 and BL, C4 and C2, and C2 and BL were statistically significant with p < 0.001**.** Radiomic features from BL DCE MRI and DWI images showed similar yet consistently worse performance in univariate analysis; all AUCs were less than 0.63 for DCE MRI and less than 0.65 for DWI (data not shown).

### Performance of the radiomic models by multivariate analysis

**Figure 1:**
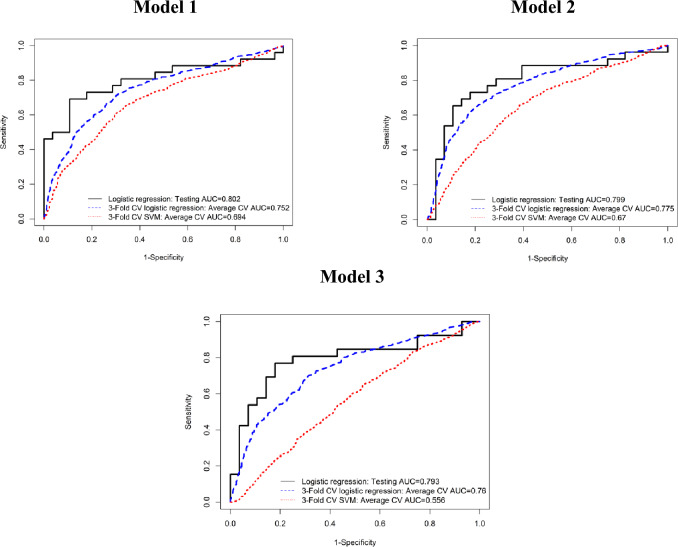
ROC curves of the three best-performing radiomic models for pCR prediction. Testing cohort using logistic regression with elastic net (black solid line). Entire cohort using threefold cross validation logistic regression with elastic net (blue dashed line), and threefold cross validation support vector machine (SVM) with linear kernel (red dotted line). FPR, false positive rate.

Thirty-six models from multivariate analysis based on both DCE MRI and DWI had AUC > 0.7 in both the training and testing sets (*p* < 0.003), and 18 of these models had AUC > 0.75 in both the training and testing sets (*p* < 0.001). The 3 top-performing models combined radiomic features corresponding to relative difference between BL and C2, absolute difference between BL and C4, and C4 features from DCE MRI and DWI images and all had AUC > 0.79 in both the training and testing sets (Table 2, Fig. 1). The best model combined 35 radiomic features corresponding to relative difference between BL and C2 and had AUC = 0.91 in the training set and AUC = 0.80 in the testing set (*p* < 0.001). The second-best model combined 44 radiomic features of absolute difference between BL and C4 and had AUC = 0.90 in the training set and AUC = 0.80 in the testing set (*p* < 0.001). The third-best model combined 18 radiomic features from C4 and the absolute difference between BL and C4 and had AUC = 0.82 in the training set and AUC = 0.79 in the testing set (*p* < 0.001).

**Table 2:** Top-performing multivariate models for pCR prediction.

Model	Variable			Training (N = 109)			Testing (N = 54)			*P* value
	Time point	DCE	DWI	AUC	Accuracy	AUC CI	AUC	Accuracy	AUC CI	
**1**	RD, C2/BL	Minimum Maximum 5 GLCM features	1st percentile 95th percentile Minimum Kurtosis 24 GLCM features	0.91	0.84	0.850—0.959	0.80	0.76	0.674—0.930	< 0.001
**2**	AD, C4/BL	5th percentile 95th percentile 11 GLCM features	Standard deviation 95th percentile Mean 28 GLCM features	0.90	0.80	0.847—0.960	0.80	0.76	0.671—0.928	< 0.001
**3**	C4	10 GLCM features	3 GLCM features	0.82	0.71	0.744—0.899	0.79	0.76	0.662—0.923	< 0.001
	AD, C4/BL	–	5 GLCM							

AD, Absolute difference; RD, Relative difference; BL, Baseline; C2, After 2 cycles of NAST; C4, After 4 cycles of NAST; GLCM, Gray level co-occurrence matrix; AUC, Area under the receiver operating characteristic (ROC) curve; CI 95% Confidence interval.

The threefold cross-validation without stratified splitting had similar and comparable AUC to training/testing-based AUC. The first model had an AUC of 0.75, the second model had an AUC of 0.78, and the third model had an AUC of 0.76 (Fig. 1). As a comparison to the elastic net-based logistic regression, support vector machine (SVM) with a linear, radial basis function (RBF), or Gaussian kernel, yielded AUC of <  = 0.72 for all the 3 models. Thus, the logistic model had a better performance than SVM (Supplementary Table 4).

### Inter-reader and intrareader agreement

Pearson correlation analysis for features extracted from DCE images showed that both GLCM features and first-order features had excellent correlation between readers and that GLCM features had better correlations than first-order features. The Pearson correlation coefficient was > 0.8 for 25 of 30 (83%) first-order features (7 BL, 10 C2, and 8 C4), 18 of 30 (60%) absolute difference first-order features (6 C2/BL, 7 C4/BL, and 5 C4/C2), 20 of 30 (67%) relative difference first-order features (8 C2/BL, 8 C4/BL, and 4 C4/C2), 300 of 300 (100%) GLCM features, 300 of 300 (100%) absolute difference GLCM features, and 271 of 300 (90.3%) relative difference GLCM features. As for the ratio of inter-reader variance to intrareader variance for the first-order features, the mean value was 0.006, the median value was 0, the range was 0 to 0.077, and the standard deviation was 0.016, indicating high agreement between the 2 readers. The ratios of inter-reader variance to intrareader variance for all of the GLCM features were zeros rounding to the nearest ten thousandth, indicating little variability between the 2 readers for any of the GLCM features. Overall, inter-reader variability was only minimally higher than intrareader variability. Furthermore, pCR prediction models extracted from DCE images showed similar AUC values for the 2 readers (Supplementary Table 3).

## Discussion

In this study, we investigated the performance of radiomic signatures derived from multiparametric MRI images obtained early during NAST for prediction of treatment response in patients with TNBC. Top performance was achieved by models that included radiomic features from DCE MRI and DWI images acquired at C4, changes between BL and C2, and changes between BL and C4. These models had high inter-reader agreement and therefore could potentially be used as a quantitative tool to predict the pCR to NAST.

Among patients with TNBC, those with a pCR to NAST have a much better prognosis than those with a non-pCR. A method to accurately predict the response to NAST early in the treatment course would allow patients predicted to have a pCR to continue the treatment or have treatment de-escalation and patients predicted to have a non-pCR to be switched to different therapy or offered novel targeted trials.

Results from several studies showed that radiomic features may be useful for breast tumor detection and for prediction of treatment response, tumor molecular subtype, and axillary lymph node metastases in patients with breast cancer^25–31^. Combined application of different imaging sequences was found to be superior to use of a single sequence in prediction of pCR after NAST^32–34^.

A study by Chen et al. included 91 patients with different breast cancer molecular subtypes and 396 radiomic texture features from multiparametric MRI. The models based on pretreatment DCE MRI and ADC data were able to predict pCR with greater accuracy than the models based on either DCE MRI or ADC alone^33^. Xiong et al. analyzed 125 breast cancer patients with different hormonal subtypes who underwent multiparametric MRI before receiving NAST. Their combined model incorporating a radiomic signature constructed with DCE MRI, DWI, and T2-weighted imaging features, HER2 status and Ki67 index was able to identify patients who would be insensitive to NAST before treatment, with an AUC of 0.935 in the independent validation cohort^35^. Bian et al. found that multiparametric models of radiomic signatures based on pretreatment T2-weighted, DWI, and DCE MRI images of 152 breast cancer patients had the best performance in predicting pCR with AUC 0.91–0.93 for all molecular subtypes of breast cancer^32^. In contrast, our study evaluated MRI radiomic features at multiple time points for pCR prediction and only included patients with TNBC. Clinical data and T2-weighted images were not included in our analysis and might have further improved the performance of our model.

Li et al. demonstrated that a combined analysis of multiparametric quantitative MRI measurements obtained after the first cycle of NAST allowed the prediction of pCR in 33 breast cancer patients with an AUC of 0.88 and was superior to DCE MRI or DWI alone with an AUC of 0.76 and 0.82, respectively^34^.

In a retrospective study, Liu et al. developed and validated radiomic models combining both multiparametric MRI (T2-weighted imaging, DWI, and contrast-enhanced T1-weighted imaging) characteristics of the tumor and clinical information on pretreatment MRI to predict pCR to NAST in patients with all breast cancer subtypes, with AUC of 0.86^36^.

Our study differs from previous radiomic analysis studies for prediction of response to NAST in that our models included radiomic features from a combination of DCE MRI and DWI sequences acquired at multiple time points early during the neoadjuvant treatment course (after two and four cycles of NAST), thus potentially capturing important temporal changes in tumor biology. Only patients with aggressive TNBC molecular subtype in the prospective ARTEMIS clinical trial were included in our study, while most of the previous studies were based on pretreatment MRI features from patients with all breast cancer subtypes based mainly on retrospective analyses^32,33,35,36^. To best of our knowledge, our study included the largest reported group of TNBC patients undergoing NAST with a validation in the independent testing cohort, with the best performing multivariate models achieving a high AUC of 0.8. Pretreatment multiparametric radiomic models were not able to predict pCR with reliable accuracy in our study, even though previously published reports showed high AUC for pCR prediction from baseline MRI. This difference could be attributed to the difference between the study populations.

There is increased interest in use of deep learning algorithms for prediction of response in breast cancer patients undergoing NAST. A recent study of 210 TNBC patients showed that deep learning model based on serial DCE-MRI and DWI was able to predict the breast pCR status of TNBC patients receiving NAST with good performance^22^.

Our results showed high inter-reader agreement with no difference between the 2 readers in AUC values of the pCR prediction models extracted from DCE images. Inter-reader variability was only minimally higher than intrareader variability for the first-order (histogram) features, and GLCM features showed almost no variability. Excellent inter-reader and intrareader agreement found in our study supports the possibility of using this quantitative tool for prediction of pCR in breast cancer patients receiving NAST.

Our study has some limitations. It was conducted at a single institution and thus is susceptible to selection bias. However, the MRI acquisition, core-needle biopsy specimen collection, and pathologic assessment were performed according to standard procedures, which would be expected to reduce variations. A larger study sample and prospective, ideally controlled, clinical trial in collaboration with other institutions is necessary to validate our results and provide additional information about the role of radiomics in breast cancer patients undergoing NAST. Finally, inter-reader agreement in tumor segmentation and its impact on predictive performance was assessed only for DCE images, not for DWI images. Our proposed model could be extended by including additional data, such as T2-weighted imaging and clinicopathological information, or addition of the deep learning algorithms, to further improve prediction.

## Methods

### Study cohort selection

**Figure 2:**
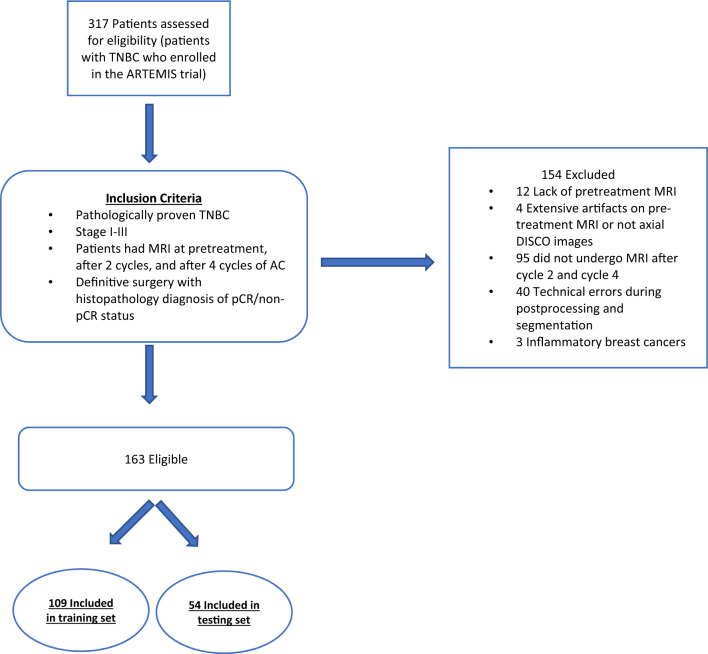
Patient inclusion and exclusion criteria.

Our study population consisted of 163 patients who were enrolled from May 2018 to July 2021 in prospective clinical trial in patients with stage I-III TNBC, “A Randomized TNBC Enrolling Trial to Confirm Molecular Profiling Improves Survival” (ARTEMIS, NCT02276443). All the patients in the trial were from a single comprehensive cancer center and were prospectively monitored for response to NAST. The trial was approved by the Institutional Review Board of The University of Texas MD Anderson Cancer Center, and written informed consent was obtained from all patients before enrollment. The study methods were performed in accordance with the relevant guidelines and regulations. All the patients in the study reported here underwent NAST and had MRI scans at baseline, after 2 cycles of NAST, and after four cycles of NAST. Figure 2 depicts the patient inclusion and exclusion criteria for this study.

### Histopathology review

Pretreatment core-needle biopsy specimens were obtained for immunohistochemical assessment and histological typing. Estrogen receptor and progesterone receptor status were evaluated using immunohistochemical staining, and tumors were defined as negative for these receptors if fewer than 10% of invasive tumor cells exhibited positive nuclear staining for these receptors. HER2 was defined as negative per the American Society of Clinical Oncology/College of American Pathologists guidelines^37^. Upon completion of NAST, patients underwent surgical resection with assessment of residual disease by dedicated breast pathologists. pCR was defined as absence of residual invasive disease in the breast and the axillary lymph nodes.

### Neoadjuvant systemic therapy

NAST consisted of dose-dense AC (doxorubicin and cyclophosphamide) for 4 cycles followed by paclitaxel every 2 weeks for 4 cycles or weekly for 12 doses. Patients with suboptimal responses who received experimental treatment were excluded to ensure homogeneity of the cohort.

### MRI data acquisition

Breast MRI was performed with patients in the prone position using a GE 3.0-T MR750w whole body scanner (Waukesha, WI) with an 8-channel phased array bilateral breast coil. The MRI protocol consisted of axial T2-weighted series, DCE MRI series, and DWI series. The DCE series was performed using the Differential Subsampling with Cartesian Ordering (DISCO) sequence with bipolar readouts for 2-point Dixon processing to produce water-only and fat-only images for each acquired slice.

Typical scan parameters used for the DISCO acquisition were as follows: field of view = 34 × 34 cm, flip angle = 12º, repetition time = 7.6 ms, echo time 1/echo time 2 = 1.1/2.3 ms, total acquisition time = 7 min, slice thickness = 3.0 mm, slice spacing = -1.5 mm, number of acquired slices = 60–115, matrix = 320 × 320, temporal resolution = 8–15.5 s, receiver bandwidth =  ± 166.7 kHz, number of excitations = 0.69, and autocalibrating reconstruction for Cartesian imaging factor = 3. During DCE series, a single bolus of gadobutrol contrast agent (Gadovist, Bayer Health Care) was injected (0.1 mL/kg at ~ 2 mL/second followed by saline flush) after at least 1 mask phase was obtained. Serial subtraction images were generated during postprocessing. Early subtraction images relative to the mask series were generated at approximately 2.5 min after injection.

DWI images were acquired prior to DCE MRI with a reduced field-of-view sequence (FOCUS DWI). Compared to conventional DWI, FOCUS DWI allows a shorter echo train for a desired resolution, with reduced image blurring and reduced artifacts. Typical scan parameters for FOCUS DWI were as follows: TE/TR = 70/4000 ms, matrix size = 80 × 80, field of view = 16 × 16 cm^2^, number of slices = 13–16, slice thickness = 4 mm, slice gap = 0 mm, scan duration = 5 min. The b-values and corresponding numbers of signal averages were 100 (4) and either 800 (16) or 1000 (16) sec/mm^2^.

### Image preprocessing, tumor segmentation, and volume extraction

**Figure 3:**
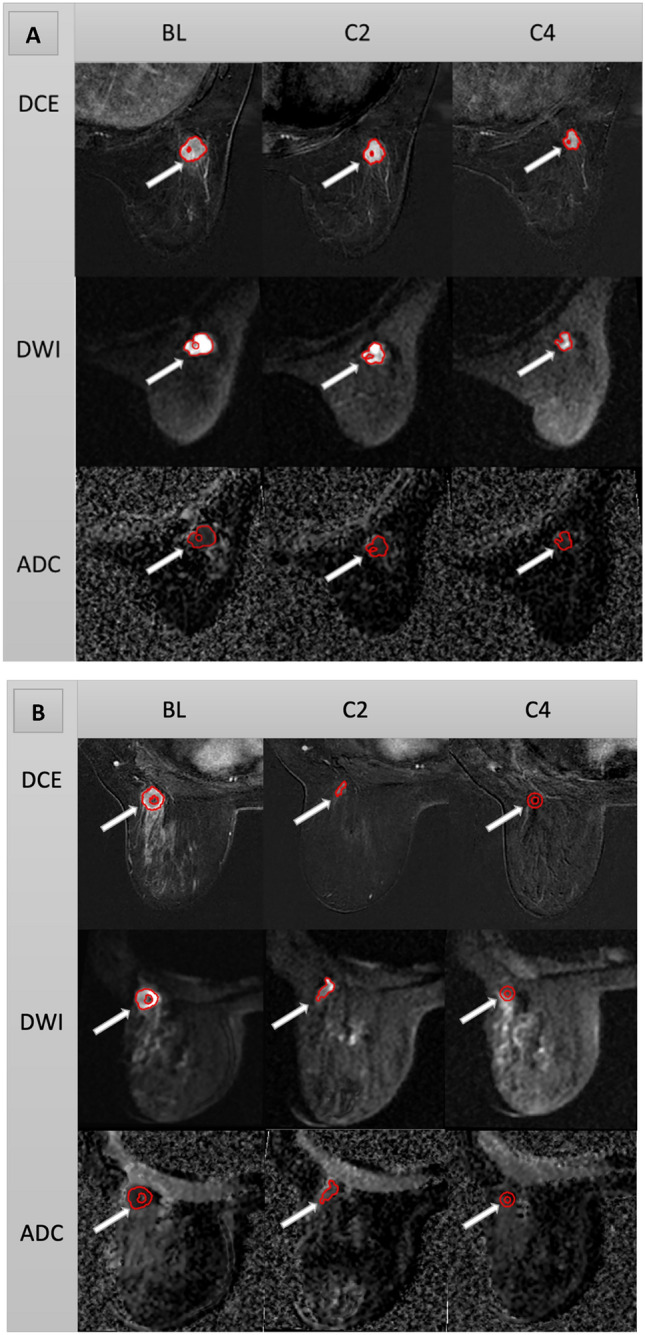
Tumor contouring. (**A**) Fifty-five-year-old woman with triple-negative invasive ductal carcinoma of the right breast. DCE, DWI and ADC images show a segmented irregular mass at 7 o’clock (arrows) that measured 2.1 × 1.9 × 1.4 cm at BL, 1.7 × 1.5 × 1.2 cm at C2, and 1.5 × 1.5 × 1 cm at C4. Histopathologic assessment at surgery revealed non-pCR. (**B**) Fifty-one-year-old woman with triple-negative invasive ductal carcinoma of the left breast. DCE, DWI and ADC images show a segmented irregular mass at 2 o’clock (arrows) that measured 3 × 2.1 × 2.1 cm at BL and had decreased in size to 1.3 × 1.2 × 0.9 cm at C2. At C4, there was complete resolution of the mass and tumor bed was segmented (arrows). Histopathologic assessment at surgery revealed pCR.

For tumor segmentation, the phase at 2.5 min after injection of contrast medium from the DISCO fast DCE MRI acquisition was selected as an early phase for segmentation. ADC maps were generated from the acquired FOCUS DWI images using a mono-exponential model. If there were multiple lesions, the largest lesion identified on DCE images was considered the index carcinoma. Tumor contouring was performed by 2 breast radiologists with 8 years (M.B.) and 5 years (R.M.) of experience who were blinded to the patient clinical outcomes and in the order of trial enrollment, on the BL, C2, and C4 images using an in-house image analysis software program (Image-I)^38,39^. Tumors were first manually defined across all slices on the 2.5-min early subtraction images and b = 800 DWI images, and all volumes of interest (VOIs) were semi-automatically segmented using the histogram thresholding in Image-I. Tumor necrotic regions and clip artifacts were segmented out of the VOIs to ensure proper phenotype border selection. If tumors were not visible on the C2 scan or the C4 scan, the tumor bed was contoured (Fig. 3). The inter-reader agreement was analyzed between the VOIs of DCE maps obtained from the 2 breast radiologists (M.B. and R.M.) following the same procedure.

### Radiomic analysis

**Figure 4:**
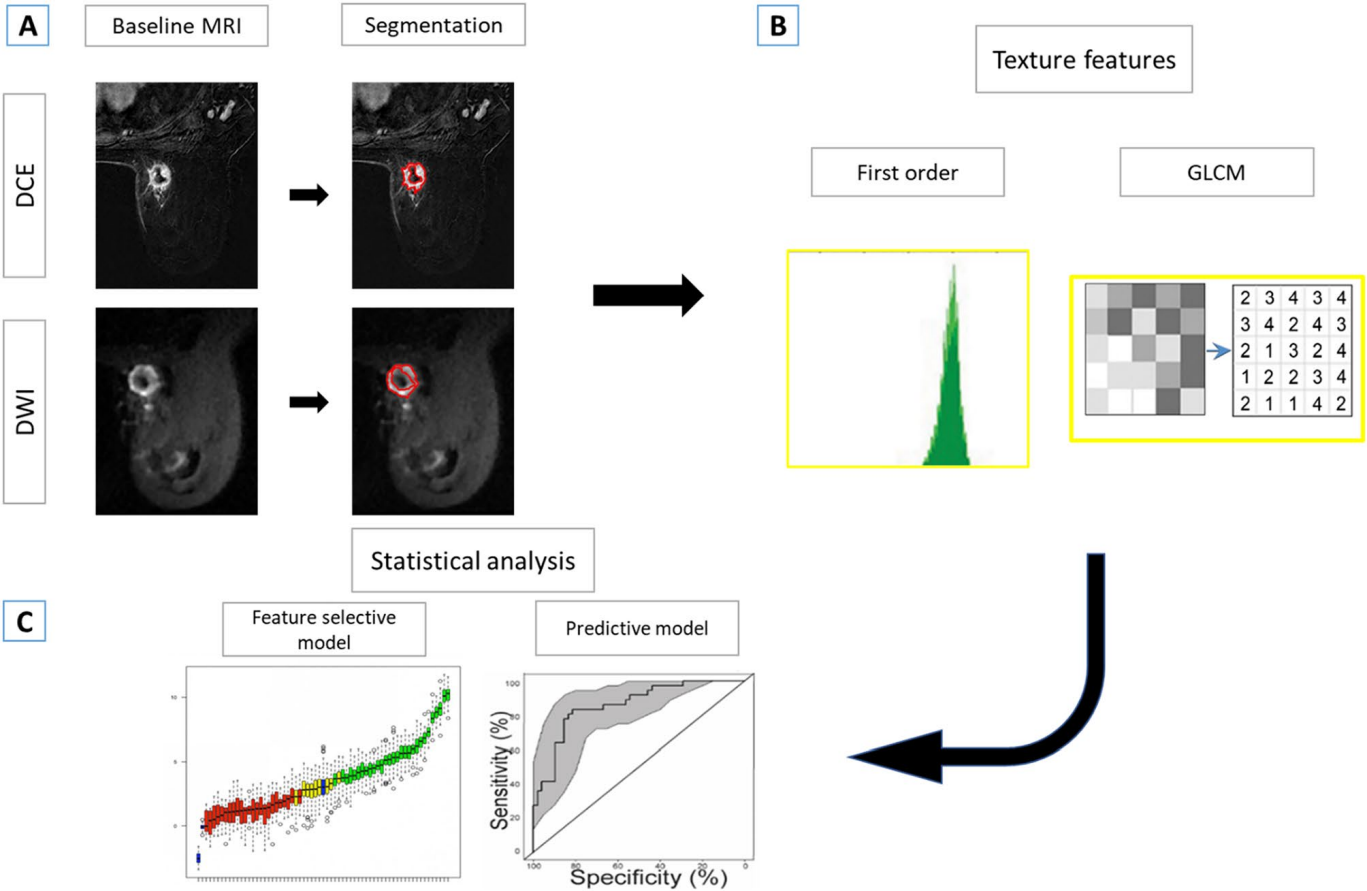
Radiomics prediction pipeline for pCR. (**A**) Segmentation process of DCE and DWI images. (**B**) Different radiomic features extraction. (**C**) Statistical analysis to search for the best model for pCR prediction.

Radiomic features were extracted using an in-house software package written in MATLAB (MathWorks Inc, Natick, MA). Within the segmented VOI, 10 first-order radiomic features (minimum, maximum, mean, standard deviation, kurtosis, skewness, and first, fifth, 95th, and 99th percentiles) and 300 GLCM radiomic features (histogram features and texture features) were extracted from the VOIs of the 2.5-min early subtraction images and b = 800 DWI images. Figure 4 shows the radiomic analysis pipeline.

### Statistical analysis

Patient clinicopathologic characteristics were summarized using frequencies, percentages, means, standard deviations, medians, minimums, and maximums. The demographic and clinical characteristics were compared between patients with pCR and non-pCR using Wilcoxon rank sum test or Fisher’s exact test.

AUC was used for univariate analysis in evaluating the performance of predicting pCR status. Logistic regression with elastic net regularization was performed for radiomic feature selection. Both regularization and mixing parameters were optimized using fivefold cross-validation based on AUC. Stratified sampling was applied to divide subjects into training and testing data. The performance of the training/testing-based logistic regression with the elastic net penalty was compared with threefold cross-validation logistic regression model with the elastic net penalty and SVM with the linear, radial basis function (RBF), or Gaussian kernel classifier.

P-value less than 0.05 was considered statistically significant. For inter-reader agreement, the Pearson correlation was computed to evaluate the linear relationship of each imaging feature between 2 readers. Wilcoxon signed-rank test was applied to test the difference between 2 readers per feature. The ratios of inter-reader to intrareader variability were calculated per feature and summarized by range, mean, and median. The statistical analysis was performed by using R software (version 4.0.3, R Foundation for Statistical Computing, Vienna, Austria) and the packages glmnet and pROC (version 1.9.1).

### Supplementary Information

Supplementary Information.

## Data Availability

Data are available from the corresponding author upon request.

## Code availability

Code is available from the corresponding author upon request.
