# Supplementary material for: Multiparametric MRI–based radiomic models for early prediction of response to neoadjuvant systemic therapy in triple-negative breast cancer
Source: Sci Rep. 2024 Jul 12;14:16073. doi: 10.1038/s41598-024-66220-9 (PMC11239818; doi:10.1038/s41598-024-66220-9)
Supplement: Supplementary file 1 — Supplementary Information. [file 41598_2024_66220_MOESM1_ESM.docx]

**Supplementary Table 1** Radiomic features with AUC>0.7 from the univariate analysis of DCE MRI data*

|  | **Training (N=109)** | | **Testing (N=54)** | |
| --- | --- | --- | --- | --- |
| **Feature** | **AUC** | **95% CI** | **AUC** | **95% CI** |
| DCE_RDC4C2_ Mean | 0.845 | 0.771-0.919 | 0.709 | 0.569-0.848 |
| DCE_RDC4BS_ Mean | 0.843 | 0.772-0.915 | 0.775 | 0.643-0.906 |
| DCE_RDC4BS_ Percentile.95 | 0.833 | 0.759-0.907 | 0.754 | 0.617-0.891 |
| DCE_RDC4BS_ Maximum | 0.831 | 0.756-0.906 | 0.747 | 0.607-0.888 |
| DCE_RDC4BS_ Percentile.99 | 0.831 | 0.756-0.906 | 0.750 | 0.612-0.888 |
| DCE_RDC4BS_ Percentile.5 | 0.826 | 0.749-0.903 | 0.805 | 0.681-0.929 |
| DCE_RDC4C2_ Percentile.5 | 0.826 | 0.747-0.905 | 0.772 | 0.647-0.897 |
| DCE_RDC4BS_ Percentile.1 | 0.818 | 0.738-0.899 | 0.810 | 0.687-0.934 |
| DCE_C4_ Mean | 0.810 | 0.730-0.891 | 0.717 | 0.573-0.861 |
| DCE_RDC4C2_ Percentile.1 | 0.805 | 0.721-0.889 | 0.760 | 0.631-0.888 |
| DCE_C4_ Percentile.5 | 0.799 | 0.715-0.883 | 0.713 | 0.568-0.858 |
| DCE_ADC4BS_ Percentile.99 | 0.796 | 0.713-0.879 | 0.731 | 0.591-0.871 |
| DCE_RDC4C2_ Percentile.99 | 0.792 | 0.707-0.877 | 0.702 | 0.557-0.846 |
| DCE_C4_ Percentile.1 | 0.790 | 0.703-0.876 | 0.743 | 0.602-0.884 |
| DCE_ADC4BS_ Percentile.95 | 0.789 | 0.704-0.873 | 0.760 | 0.623-0.896 |
| DCE_RDC4BS_ Minimum | 0.789 | 0.703-0.874 | 0.787 | 0.653-0.921 |
| DCE_ADC4BS_ Percentile.5 | 0.788 | 0.701-0.874 | 0.813 | 0.688-0.938 |
| DCE_ADC4BS_ Mean | 0.786 | 0.700-0.872 | 0.766 | 0.630-0.903 |
| DCE_ADC4BS_ Percentile.1 | 0.785 | 0.698-0.873 | 0.812 | 0.692-0.933 |
| DCE_ADC4BS_ Maximum | 0.782 | 0.696-0.867 | 0.723 | 0.579-0.867 |

**Supplementary Table 1 (continued)** Radiomic features with AUC>0.7 from the univariate analysis of DCE MRI data*

|  | **Training (N=109)** | | **Testing (N=54)** | | |
| --- | --- | --- | --- | --- | --- |
| **Feature** | **AUC** | **95% CI** | **AUC** | **95% CI** | |
| DCE_ADC4C2_ Percentile.1 | 0.763 | 0.675-0.852 | 0.713 | | 0.573-0.853 |
| DCE_C4_ Minimum | 0.737 | 0.642-0.831 | 0.769 | | 0.634-0.905 |
| DCE_C4_ Minimum | 0.737 | 0.642-0.831 | 0.769 | | 0.634-0.905 |
| DCE_ADC4BS_ Minimum | 0.710 | 0.610-0.809 | 0.761 | | 0.621-0.901 |
| DCE_RDC2BS_ Maximum | 0.704 | 0.605-0.804 | 0.709 | | 0.567-0.851 |

AD, absolute difference; RD, relative difference.

*P<0.001 for all features.

**Supplementary Table 2** Radiomic features with AUC > 0.7 from the univariate analysis of DWI data *

|  | **Training (N=109)** | | **Testing (N=54)** | |
| --- | --- | --- | --- | --- |
| **Feature** | **AUC** | **95% CI** | **AUC** | **95% CI** |
| DWI_C2_GLCM_F124 | 0.793 | 0.708-0.877 | 0.717 | 0.575-0.859 |
| DWI_C2_GLCM_F184 | 0.793 | 0.709-0.877 | 0.721 | 0.580-0.862 |
| DWI_C2_GLCM_F244 | 0.793 | 0.708-0.877 | 0.720 | 0.579-0.861 |
| DWI_C2_GLCM_F64 | 0.791 | 0.707-0.876 | 0.716 | 0.573-0.858 |
| DWI_C2_GLCM_F4 | 0.787 | 0.702-0.873 | 0.709 | 0.565-0.852 |
| DWI_C2_GLCM_F90 | 0.762 | 0.672-0.852 | 0.701 | 0.560-0.841 |
| DWI_C2_GLCM_F229 | 0.761 | 0.672-0.849 | 0.707 | 0.566-0.849 |
| DWI_C2_GLCM_F296 | 0.760 | 0.671-0.849 | 0.716 | 0.576-0.855 |
| DWI_C2_GLCM_F170 | 0.758 | 0.668-0.849 | 0.701 | 0.560-0.841 |
| DWI_C2_GLCM_F110 | 0.755 | 0.664-0.846 | 0.720 | 0.582-0.857 |
| DWI_C2_GLCM_F149 | 0.754 | 0.663-0.844 | 0.705 | 0.565-0.844 |
| DWI_C2_GLCM_F288 | 0.754 | 0.664-0.844 | 0.717 | 0.577-0.857 |
| DWI_C2_GLCM_F228 | 0.750 | 0.659-0.84 | 0.718 | 0.579-0.858 |
| DWI_C2_GLCM_F298 | 0.750 | 0.659-0.842 | 0.705 | 0.561-0.848 |
| DWI_C2_GLCM_F148 | 0.746 | 0.655-0.837 | 0.710 | 0.571-0.850 |
| DWI_C2_GLCM_F238 | 0.744 | 0.652-0.836 | 0.707 | 0.563-0.852 |
| DWI_C2_GLCM_F169 | 0.740 | 0.648-0.832 | 0.712 | 0.572-0.851 |
| DWI_C2_GLCM_F178 | 0.739 | 0.646-0.832 | 0.702 | 0.558-0.846 |
| DWI_C2_GLCM_F247 | 0.738 | 0.646-0.831 | 0.729 | 0.591-0.867 |
| DWI_C2_GLCM_F250 | 0.738 | 0.646-0.831 | 0.729 | 0.591-0.867 |

**Supplementary Table 2 (continued)** Radiomic features with AUC > 0.7 from the univariate analysis of DWI data *

|  | **Training (N=109)** | | **Testing (N=54)** | |
| --- | --- | --- | --- | --- |
| **Feature** | **AUC** | **95% CI** | **AUC** | **95% CI** |
| DWI_C2_GLCM_F168 | 0.737 | 0.644-0.829 | 0.720 | 0.580-0.859 |
| DWI_C2_GLCM_F190 | 0.733 | 0.640-0.827 | 0.723 | 0.584-0.861 |
| DWI_C2_GLCM_F187 | 0.732 | 0.639-0.826 | 0.723 | 0.584-0.862 |
| DWI_C2_GLCM_F254 | 0.724 | 0.628-0.819 | 0.721 | 0.581-0.861 |
| DWI_C2_GLCM_F130 | 0.720 | 0.624-0.816 | 0.709 | 0.568-0.849 |
| DWI_C2_GLCM_F249 | 0.718 | 0.622-0.814 | 0.721 | 0.582-0.861 |
| DWI_C2_GLCM_F127 | 0.717 | 0.621-0.813 | 0.707 | 0.566-0.848 |
| DWI_C2_GLCM_F248 | 0.715 | 0.618-0.812 | 0.721 | 0.581-0.861 |
| DWI_C2_GLCM_F194 | 0.714 | 0.617-0.811 | 0.713 | 0.571-0.855 |
| DWI_C2_GLCM_F188 | 0.702 | 0.603-0.800 | 0.707 | 0.565-0.850 |
| DWI_C2_GLCM_F256 | 0.702 | 0.604-0.800 | 0.707 | 0.565-0.849 |
| DWI_C4_GLCM_F209 | 0.771 | 0.684-0.857 | 0.797 | 0.670-0.924 |
| DWI_C4_GLCM_F229 | 0.770 | 0.683-0.856 | 0.810 | 0.684-0.937 |
| DWI_C4_GLCM_F296 | 0.767 | 0.680-0.854 | 0.808 | 0.681-0.935 |
| DWI_C4_GLCM_F288 | 0.764 | 0.676-0.852 | 0.809 | 0.683-0.935 |
| DWI_C4_GLCM_F268 | 0.760 | 0.672-0.849 | 0.787 | 0.657-0.917 |
| DWI_C4_GLCM_F228 | 0.759 | 0.670-0.847 | 0.799 | 0.672-0.927 |
| DWI_C4_GLCM_F269 | 0.759 | 0.670-0.848 | 0.750 | 0.614-0.886 |
| DWI_C4_GLCM_F208 | 0.757 | 0.669-0.846 | 0.782 | 0.650-0.913 |
| DWI_C4_GLCM_F236 | 0.756 | 0.667-0.845 | 0.787 | 0.656-0.918 |

**Supplementary Table 2 (continued)** Radiomic features with AUC > 0.7 from the univariate analysis of DWI data *

|  | **Training (N=109)** | | **Testing (N=54)** | |
| --- | --- | --- | --- | --- |
| **Feature** | **AUC** | **95% CI** | **AUC** | **95% CI** |
| DWI_C4_GLCM_F289 | 0.756 | 0.667-0.845 | 0.784 | 0.652-0.917 |
| DWI_C4_GLCM_F244 | 0.755 | 0.665-0.846 | 0.791 | 0.664-0.919 |
| DWI_C4_GLCM_F276 | 0.755 | 0.666-0.844 | 0.764 | 0.629-0.899 |
| DWI_C4_GLCM_F184 | 0.754 | 0.663-0.845 | 0.791 | 0.664-0.919 |
| DWI_C4_GLCM_F124 | 0.753 | 0.662-0.844 | 0.790 | 0.662-0.917 |
| DWI_C4_GLCM_F64 | 0.748 | 0.656-0.840 | 0.786 | 0.658-0.914 |
| DWI_C4_GLCM_F149 | 0.748 | 0.657-0.839 | 0.783 | 0.653-0.913 |
| DWI_C4_GLCM_F125 | 0.742 | 0.650-0.834 | 0.790 | 0.660-0.919 |
| DWI_C4_GLCM_F185 | 0.742 | 0.650-0.834 | 0.793 | 0.663-0.922 |
| DWI_C4_GLCM_F245 | 0.742 | 0.650-0.834 | 0.790 | 0.659-0.921 |
| DWI_C4_GLCM_F4 | 0.741 | 0.648-0.833 | 0.776 | 0.646-0.907 |
| DWI_C4_GLCM_F148 | 0.741 | 0.650-0.833 | 0.761 | 0.625-0.897 |
| DWI_C4_GLCM_F169 | 0.739 | 0.647-0.831 | 0.782 | 0.651-0.913 |
| DWI_C4_GLCM_F218 | 0.739 | 0.646-0.833 | 0.776 | 0.644-0.908 |
| DWI_C4_GLCM_F278 | 0.739 | 0.645-0.832 | 0.779 | 0.648-0.910 |
| DWI_C4_GLCM_F65 | 0.738 | 0.646-0.830 | 0.787 | 0.658-0.916 |
| DWI_C4_GLCM_F90 | 0.738 | 0.644-0.831 | 0.802 | 0.679-0.925 |
| DWI_C4_GLCM_F216 | 0.737 | 0.645-0.829 | 0.750 | 0.612-0.888 |
| DWI_C4_GLCM_F247 | 0.735 | 0.642-0.828 | 0.783 | 0.652-0.914 |
| DWI_C4_GLCM_F250 | 0.735 | 0.642-0.828 | 0.784 | 0.654-0.915 |

**Supplementary Table 2 (continued)** Radiomic features with AUC > 0.7 from the univariate analysis of DWI data *

|  | **Training (N=109)** | | **Testing (N=54)** | |
| --- | --- | --- | --- | --- |
| **Feature** | **AUC** | **95% CI** | **AUC** | **95% CI** |
| DWI_C4_GLCM_F168 | 0.733 | 0.641-0.826 | 0.771 | 0.637-0.904 |
| DWI_C4_GLCM_F158 | 0.731 | 0.636-0.825 | 0.772 | 0.639-0.905 |
| DWI_C4_GLCM_F298 | 0.730 | 0.635-0.824 | 0.782 | 0.651-0.912 |
| DWI_C4_GLCM_F5 | 0.727 | 0.634-0.821 | 0.769 | 0.636-0.902 |
| DWI_C4_GLCM_F110 | 0.726 | 0.631-0.821 | 0.812 | 0.690-0.933 |
| DWI_C4_GLCM_F150 | 0.724 | 0.629-0.819 | 0.773 | 0.646-0.901 |
| DWI_C4_GLCM_F187 | 0.724 | 0.630-0.819 | 0.772 | 0.639-0.904 |
| DWI_C4_GLCM_F190 | 0.724 | 0.630-0.818 | 0.773 | 0.641-0.906 |
| DWI_C4_GLCM_F238 | 0.723 | 0.628-0.818 | 0.783 | 0.652-0.914 |
| DWI_C4_GLCM_F178 | 0.718 | 0.621-0.814 | 0.775 | 0.642-0.908 |
| DWI_C4_GLCM_F254 | 0.718 | 0.622-0.814 | 0.743 | 0.603-0.883 |
| DWI_C4_GLCM_F98 | 0.714 | 0.617-0.810 | 0.750 | 0.614-0.886 |
| DWI_C4_GLCM_F170 | 0.712 | 0.616-0.808 | 0.786 | 0.660-0.911 |
| DWI_C4_GLCM_F249 | 0.712 | 0.616-0.808 | 0.750 | 0.612-0.888 |
| DWI_C4_GLCM_F130 | 0.711 | 0.615-0.808 | 0.755 | 0.619-0.892 |
| DWI_C4_GLCM_F210 | 0.711 | 0.614-0.808 | 0.75 | 0.617-0.883 |
| DWI_C4_GLCM_F248 | 0.711 | 0.614-0.808 | 0.74 | 0.601-0.880 |
| DWI_C4_GLCM_F117 | 0.710 | 0.613-0.807 | 0.779 | 0.650-0.908 |
| DWI_C4_GLCM_F127 | 0.708 | 0.611-0.805 | 0.74 | 0.601-0.879 |
| DWI_C4_GLCM_F194 | 0.708 | 0.611-0.805 | 0.72 | 0.577-0.863 |

**Supplementary Table 2 (continued)** Radiomic features with AUC > 0.7 from the univariate analysis of DWI data *

|  | **Training (N=109)** | | **Testing (N=54)** | |
| --- | --- | --- | --- | --- |
| **Feature** | **AUC** | **95% CI** | **AUC** | **95% CI** |
| DWI_C4_GLCM_F270 | 0.708 | 0.611-0.805 | 0.735 | 0.598-0.872 |
| DWI_C4_GLCM_F88 | 0.706 | 0.610-0.803 | 0.714 | 0.570-0.858 |
| DWI_C4_GLCM_F156 | 0.706 | 0.610-0.803 | 0.718 | 0.575-0.861 |
| DWI_C4_GLCM_F177 | 0.706 | 0.609-0.803 | 0.758 | 0.627-0.890 |
| DWI_C4_GLCM_F118 | 0.705 | 0.607-0.802 | 0.760 | 0.624-0.896 |
| DWI_C4_GLCM_F89 | 0.702 | 0.605-0.799 | 0.724 | 0.583-0.865 |
| DWI_C4_GLCM_F176 | 0.701 | 0.604-0.799 | 0.736 | 0.596-0.877 |
| DWI_C4_GLCM_F230 | 0.701 | 0.604-0.799 | 0.766 | 0.636-0.897 |
| DWI_C4_GLCM_F263 | 0.700 | 0.601-0.798 | 0.721 | 0.583-0.859 |
| DWI_ADC2BS_GLCM_F238 | 0.765 | 0.673-0.858 | 0.706 | 0.566-0.846 |
| DWI_ADC2BS_GLCM_F178 | 0.757 | 0.664-0.850 | 0.702 | 0.561-0.843 |
| DWI_ADC4BS_GLCM_F238 | 0.723 | 0.627-0.819 | 0.776 | 0.650-0.902 |
| DWI_ADC4BS_GLCM_F178 | 0.717 | 0.620-0.814 | 0.764 | 0.634-0.893 |
| DWI_ADC4BS_GLCM_F110 | 0.711 | 0.611-0.810 | 0.761 | 0.633-0.889 |
| DWI_ADC4BS_GLCM_F170 | 0.700 | 0.597-0.803 | 0.709 | 0.569-0.849 |
| DWI_ADC4BS_GLCM_F218 | 0.700 | 0.602-0.799 | 0.724 | 0.584-0.864 |
| DWI_RDC2BS_GLCM_F298 | 0.745 | 0.653-0.837 | 0.705 | 0.560-0.850 |
| DWI_RDC2BS_GLCM_F289 | 0.735 | 0.642-0.829 | 0.703 | 0.561-0.846 |
| DWI_RDC2BS_GLCM_F290 | 0.717 | 0.620-0.813 | 0.705 | 0.564-0.846 |
| DWI_RDC4BS_GLCM_F289 | 0.745 | 0.653-0.837 | 0.797 | 0.663-0.931 |

**Supplementary Table 2 (continued)** Radiomic features with AUC > 0.7 from the univariate analysis of DWI data *

|  | **Training (N=109)** | | **Testing (N=54)** | |
| --- | --- | --- | --- | --- |
| **Feature** | **AUC** | **95% CI** | **AUC** | **95% CI** |
| DWI_RDC4BS_GLCM_F278 | 0.721 | 0.625-0.817 | 0.764 | 0.630-0.898 |
| DWI_RDC4BS_GLCM_F269 | 0.719 | 0.624-0.814 | 0.769 | 0.635-0.904 |
| DWI_RDC4BS_GLCM_F298 | 0.719 | 0.623-0.815 | 0.772 | 0.639-0.905 |
| DWI_RDC4BS_GLCM_F250 | 0.706 | 0.607-0.804 | 0.735 | 0.600-0.869 |
| DWI_RDC4BS_GLCM_F247 | 0.705 | 0.606-0.804 | 0.734 | 0.599-0.868 |
| DWI_RDC4BS_GLCM_F190 | 0.701 | 0.602-0.800 | 0.713 | 0.574-0.852 |

AD, absolute difference; RD, relative difference; F, feature.

*P<0.001 for all features.

**Supplementary Table 3** AUC values for reader 1 and reader 2 for pCR prediction radiomic models extracted from DCE images

|  | | | **Reader 1** | | | **Reader 2** | | |
| --- | --- | --- | --- | --- | --- | --- | --- | --- |
| **Model** | **No. in training set** | **No. in testing set** | **AUC in training set** | **AUC in testing set** | **p value** | **AUC in training set** | **AUC in testing set** | **p value** |
| C2_FO | 108 | 53 | 0.784 | 0.723 | 0.002 | 0.738 | 0.667 | 0.019 |
| C4_FO | 108 | 53 | 0.819 | 0.723 | 0.002 | 0.831 | 0.723 | 0.002 |
| ADC4BS_FO | 108 | 53 | 0.797 | 0.779 | <0.001 | 0.789 | 0.764 | <0.001 |
| RDC2BS_FO | 108 | 53 | 0.790 | 0.731 | 0.002 | 0.699 | 0.724 | 0.002 |
| RDC4BS_FO | 108 | 53 | 0.876 | 0.790 | <0.001 | 0.834 | 0.783 | <0.001 |
| ADC4BS_FO_GLCM | 108 | 53 | 0.795 | 0.766 | <0.001 | 0.792 | 0.766 | <0.001 |
| RDC4BS_FO_GLCM | 108 | 53 | 0.865 | 0.767 | <0.001 | 0.831 | 0.773 | <0.001 |
| RDC4C2_FO_GLCM | 108 | 53 | 0.845 | 0.717 | 0.003 | 0.795 | 0.714 | 0.003 |
| BS_C2_FO | 108 | 53 | 0.836 | 0.746 | <0.001 | 0.773 | 0.674 | 0.015 |
| BS_C2_ADC2BS_RDC2BS_FO | 108 | 53 | 0.857 | 0.754 | <0.001 | 0.793 | 0.757 | <0.001 |
| BS_C4_FO | 108 | 53 | 0.868 | 0.769 | <0.001 | 0.869 | 0.746 | <0.001 |
| BS_C4_ADC4BS_RDC4BS_FO_GLCM | 108 | 53 | 0.848 | 0.777 | <0.001 | 0.829 | 0.774 | <0.001 |
| BS_C4_ADC4BS_RDC4BS_FO | 108 | 53 | 0.902 | 0.786 | <0.001 | 0.884 | 0.774 | <0.001 |
| BS_C2_C4_FO_GLCM | 108 | 53 | 0.808 | 0.734 | 0.002 | 0.883 | 0.684 | 0.011 |
| BS_C2_C4_FO | 108 | 53 | 0.945 | 0.751 | <0.001 | 0.870 | 0.753 | <0.001 |
| BS_C2_C4_ADC2BS_RDC2BS_ADC4BS_RDC4BS_ADC4C2_RDC4C2_FO_GLCM | 108 | 53 | 0.864 | 0.771 | <0.001 | 0.835 | 0.776 | <0.001 |
| BS_C2_C4_ADC2BS_RDC2BS_ADC4BS_RDC4BS_ADC4C2_RDC4C2_FO | 108 | 53 | 0.953 | 0.740 | 0.001 | 0.895 | 0.771 | <0.001 |

AD, absolute difference; RD, relative difference; FO, first order

**Supplementary Table 4** Linear, radial basis function (RBF) and Gaussian kernels for the SVM

| **Model** | **Classifier** | **Cross-validation AUC** |
| --- | --- | --- |
| ADC4BS_FO_GLCM | SVM linear | 0.67 |
|  | SVM RBF | 0.70 |
|  | SVM Gaussian | 0.70 |
|  | Elastic net | 0.78 |
| RDC2BS_FO_GLCM | SVM linear | 0.70 |
|  | SVM RBF | 0.71 |
|  | SVM Gaussian | 0.72 |
|  | Elastic net | 0.75 |
| BS_C4_ADC4BS_RDC4BS_GLCM | SVM linear | 0.56 |
|  | SVM RBF | 0.66 |
|  | SVM Gaussian | 0.58 |
|  | Elastic net | 0.76 |

SVM, support vector machine; RBF, radial basis function
